# Supplementary material for: Eco-Friendly OSN Membranes Based on Alginate Salts with Variable Nanofiltration Properties
Source: Membranes (Basel). 2023 Feb 17;13(2):244. doi: 10.3390/membranes13020244 (PMC9964380; doi:10.3390/membranes13020244)
Supplement: Supplementary file 1 [file membranes-13-00244-s001.zip › membranes-2149317-supplementary.pdf]

membranes

Supplementary material

Eco-Friendly OSN Membranes Based on Alginate Salts with Variable Nanofiltration Properties

Evgenia Dmitrieva, Alisa Raeva, Daria Razlataya and Tatyana Anokhina *

A.V.Topchiev Institute of Petrochemical Synthesis RAS, Leninsky pr. 29, 119991 Moscow, Russia;
* Correspondence: tsanokhina@ips.ac.ru; Tel.: +7-(495)-647-5927 (ext. 202)

Supplementary S1. Dependence of the Rejection of Alginate Membranes on the Charge of Model Substances

All data on the membrane retention coefficient in the article "Eco-friendly OSN membranes for based on alginate salts with variable nanofiltration properties" refer to negatively charged model dyes. Retention coefficients for other types of substances: neutral and positively charged are presented in Table S1.

Table S1. The rejection of substances of different charge and molecular weight by a composite membrane with a selective layer of calcium alginate.

Dye	Charge	*MW*, g/mol	R_{DMF}	R_{EtOH}
Solvent Blue 35	Neutral	350	53	12
Orange (II)	Negative	350	55	10
Remazol Brilliant Blue R	Negative	626	87	65
Safranine O	Positive	350	18	8
Victoria Blue B	Positive	506	30	7

In the course of our research, it was found that the retention capacity of alginates for positively charged substances was extremely low. Therefore, we did not include them in the main text of the work. Perhaps this is due to the presence of sorption of these substances into the polymer matrix when the membranes are kept in organic solutions (Figure S1).

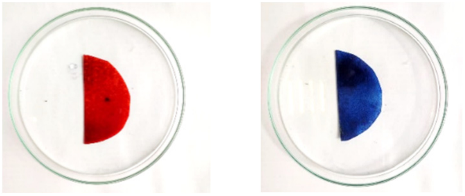

Safranine O Victoria Blue B

Figure S1. Sorption of positively charged dyes by calcium alginate from ethanol.

The membrane based on calcium alginate has approximately the same rejection of non-sorbing substances (Table 1). R of negative Orange (II) and neutral Solvent Blue 35 with the same molecular weight (350 g·mol^{-1}) is almost the same and amounts to R = 53-55% from DMF and R = 10–12% from ethanol. At the same time, the rejection of the positively charged dye Safranine O with the same molar mass is only 18 and 8% from DMF and ethanol, respectively.

Supplementary S2. Effect of Filtration on Membrane Performance.

This paper presents the filtration of organic solutions through membranes with a selective layer of calcium alginate for 24 hours. The text of the article describes the change in filtration characteristics.

At the same time, the filtration of organic solutions does not cause changes in the membrane morphology. Alginate membranes are dense selective layers with no visible porosity (Figure S2).

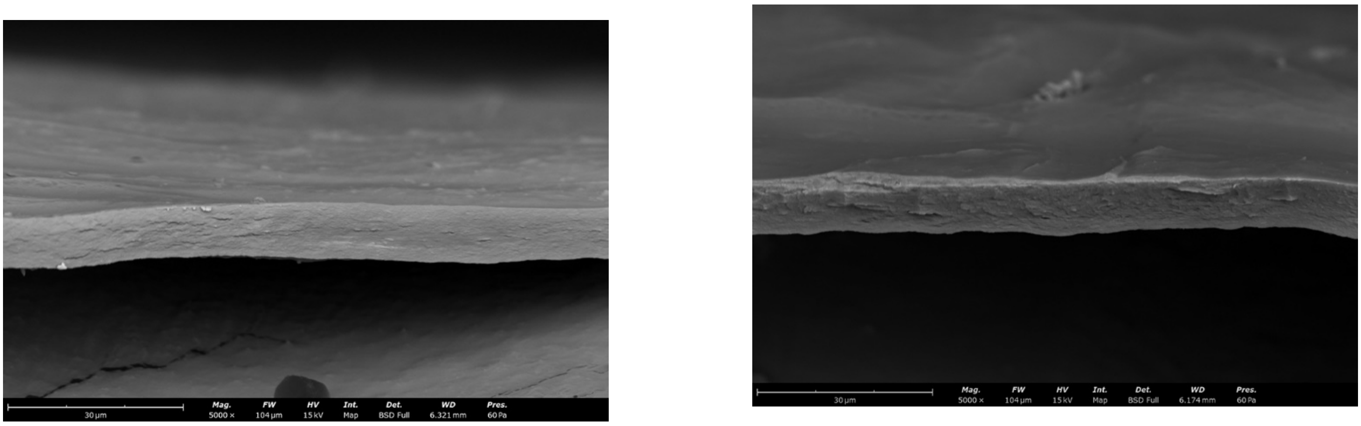

A B

Figure S2. SEM images calcium alginate before (**A**) and after (**B**) filtration EtOH + Remazol Brilliant Blue R.

During filtration, mechanical deformation and changes under the influence of membrane pressure do not occur. Figure S2 shows that there are no changes, including in thickness, before and after filtering.

As already mentioned in Supplementary 1, the filtration of solutions of negatively charged dyes in organic solvents does not lead to a visible change in the membranes and a clear adsorption of the dye on their surface. This was confirmed by a separate study on the exposure of membranes to dye solutions in organic solvents, during which the optical density of the solutions practically did not change. In the process of filtration, there is no leaching of metals from the membrane matrix. Thus, according to the EDX data, the concentration of iron before filtration is 24.9%, after filtration 25.0%.

Thus, it can be argued that the obtained membranes are stable and are not subject to significant changes during the filtration of organic solutions.
